# Predicting probability of tolerating discrete amounts of peanut protein in allergic children using epitope‐specific IgE antibody profiling

**DOI:** 10.1111/all.15477

**Published:** 2022-08-17

**Authors:** Maria Suprun, Paul Kearney, Clive Hayward, Heather Butler, Robert Getts, Scott H. Sicherer, Paul J. Turner, Dianne E. Campbell, Hugh A. Sampson

**Affiliations:** ^1^ Icahn School of Medicine at Mount Sinai New York New York USA; ^2^ AllerGenis LLC Hatfield Pennsylvania USA; ^3^ National Heart & Lung Institute, Imperial College London England UK; ^4^ The University of Sydney Sydney New South Wales Australia

**Keywords:** allergy diagnosis, CTD, DBPCFC, epitope, IgE, OFC, peanut allergy

## Abstract

**Background:**

IgE‐epitope profiling can accurately diagnose clinical peanut allergy.

**Objective:**

We sought to determine whether sequential (linear) epitope‐specific IgE (ses‐IgE) profiling can provide probabilities of tolerating discrete doses of peanut protein in allergic subjects undergoing double‐blind, placebo‐controlled food challenges utilizing PRACTALL dosing.

**Methods:**

Sixty four ses‐IgE antibodies were quantified in blood samples using a bead‐based epitope assay. A pair of ses‐IgEs that predicts Cumulative Tolerated Dose (CTD) was determined using regression in 75 subjects from the discovery cohort. This epitope‐based predictor was validated on 331 subjects from five independent cohorts (ages 4–25 years). Subjects were grouped based on their predicted values and probabilities of reactions at each CTD threshold were calculated.

**Results:**

In discovery, an algorithm using two ses‐IgE antibodies was correlated with CTDs (rho = 0.61, *p* < .05); this correlation was 0.51 (*p* < .05) in validation. Using the ses‐IgE‐based predictor, subjects were assigned into “high,” “moderate,” or “low” dose‐reactivity groups. On average, subjects in the “high” group were four times more likely to tolerate a specific dose, compared with the “low” group. For example, predicted probabilities of tolerating 4, 14, 44, and 144 or 444 mg in the “low” group were 92%, 77%, 53%, 29%, and 10% compared with 98%, 95%, 94%, 88%, and 73% in the “high” group.

**Conclusions:**

Accurate predictions of food challenge thresholds are complex due to factors including limited responder sample sizes at each dose and variations in study‐specific challenge protocols. Despite these limitations, an epitope‐based predictor was able to accurately identify CTDs and may provide a useful surrogate for peanut challenges.

## Graphical abstract

This study evaluates whether ses‐IgE profiling can predict probability of tolerating discrete doses of peanut protein in allergic subjects.There is a moderate negative correlation between the number of IgE epitopes and CTDs. The pair of ses‐IgE (Ara h 2_008 and Ara h 3_100) provide the best prediction of CRD. On average, subjects in the high‐dose reactivity group are 4 times more likely to tolerate a specific dose, compared to the low‐dose group.Abbreviations: CRD, cumulative reactive dose; CTD, cumulative tolerated dose; ses‐IgE, sequential (linear) epitope‐specific immunoglobulin E

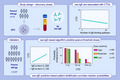

**Abbreviations**
BBEA: bead‐based epitope assay
CI: confidence interval
CRD: cumulative reactive dose
CTD: cumulative tolerated dose
DBPCFC: double‐blind, placebo‐controlled food challenge
ED: eliciting dose
IgE: immunoglobulin E
LOD: limit of detection
MFI: median fluorescence intensity
NEG: negative control sample
OFC: oral food challenge
OIT: oral immunotherapy
POS: positive control sample
Ses‐IgE: sequential epitope‐specific IgE
SPT: skin prick test

## 1 INTRODUCTION

Accurate diagnosis of food allergy is essential for both minimizing the risk of allergic reactions and eliminating unnecessary dietary restrictions. With a clear‐cut history of allergic reactions following the ingestion of specific foods, the diagnosis is relatively straightforward, while the absence of such history could lead to a challenging multi‐step diagnostic process.

Allergic sensitization can be confirmed by measuring food protein‐specific IgE by either skin prick test (SPT) wheal diameters or serum IgE levels. For peanut, an SPT wheal ≥3 mm or peanut‐specific IgE ≥0.35 kU_A_/L is considered a positive result.^1^ These cutoffs have high sensitivity but poor specificity for clinical reactivity, which could result in sensitized but clinically non‐reactive individuals being incorrectly diagnosed with peanut allergy.^2^, ^3^, ^4^ Higher cutoff values have been proposed to improve specificity and reduce the false positive rate, but with a trade‐off of lower sensitivity.^4^ When SPT and IgE testing are equivocal, further evaluation is warranted. For peanut, component‐resolved diagnostics offers improved diagnostic utility, by measuring IgE levels against specific allergen components, for example, *Ara h 1* and *Ara h 2* proteins. Several studies have shown that *Ara h 2*‐specific IgE is superior to serum IgE to whole peanut in identifying peanut‐allergic subjects.^3^, ^5^, ^6^, ^7^, ^8^ In addition to correctly diagnosing peanut allergy, an evaluation of potential severity of any reaction is also important. Unfortunately, existing diagnostics (including component‐resolved diagnostics) are not predictive of severity or the threshold dose,^9^, ^10^ and many patients still require an Oral Food Challenge (OFC).^11^ OFCs are instrumental for allergy diagnosis and determining clinical reactivity, but they often cause anaphylaxis which can increase patient anxiety and are time and resource intensive.^12^ There is still an unmet need for the development of next‐generation diagnostics that offer more granular diagnostic information, potentially reducing the need for OFCs.

Our group has previously shown that IgE specific to short sequential (linear) epitopes from the *Ara h 2* allergen can identify peanut‐allergic subjects with a sensitivity and specificity >90%.^13^ Additionally, sequential epitope‐specific IgE (ses‐IgE) diversity showed a correlation with the severity of allergic reactions to peanut.^14^, ^15^ In this current work, we sought to improve allergy diagnostics using ses‐IgE profiling to predict cumulative tolerated dose (CTD) in peanut‐allergic subjects.

## 2 METHODS

### 2.1 Sample splitting and blinding

Upon receiving baseline double‐blind, placebo‐controlled food challenge (DBPCFC) data for peanut allergic subjects in BOPI (NCT02149719)^16^ and OPIA (ACTRN12617000914369) trials, patients were randomly assigned into Discovery and Validation cohorts using a 60:40 split. To ensure that patients were well represented across the two cohorts, five randomization experiments were run, where the distribution of trials (BOPI/OPIA) were compared using a Chi‐squared test. The trial that produced the most unbiased separation, defined as Chi‐squared *p*‐value closest to 1, was selected. To ensure results' validity, a blinding protocol was generated, so that laboratory and clinical data of the Validation cohort could not be integrated until the prediction algorithm was locked.

During the discovery phase, we were able to obtain additional patient samples from baseline/enrollment DBPCFC of CAFETERIA (NCT03907397), CoFAR6 (NCT01904604),^17^ and PEPITES (NCT02636699)^18^ trials. Since, the samples were obtained after the blinding protocol and randomization were documented, to adhere to the established guidelines for the clinical diagnostic test development by the National Academy of Medicine, all samples from these studies were used only in the Validation phase. The study was approved by the local Institutional Review Boards, and all the study participants provided informed consent.

### 2.2 Epitope‐specific IgE quantification

Serum samples from BOPI, OPIA, and PEPITES, and plasma from CAFETERIA and CoFAR6 were randomized across 96‐well plates using *PlateDesigner*.^*19*^ Each study was run on a different set of plates, so that discovery and validation samples were processed separately. A Bead‐Based Epitope Assay (BBEA) was carried out as described previously,^20^ quantifying IgE antibodies to 64 15‐mer sequential epitopes from three peanut proteins: *Ara h 1* (*n* = 34), *Ara h 2* (*n* = 16), *Ara h 3* (*n* = 14), with all amino acid sequences published elsewhere.^21^, ^22^ *Ara h 2* is the smallest allergen of the three proteins, with 16 epitopes providing a 33% coverage of 49 possible 15‐mer peptides; followed by 17% coverage of the *Ara h 1* epitopes (34/205) and 8% for *Ara h 3* (14/165).

Biotinylated peptides were coupled to LumAvidin microspheres (Luminex Corporation) and this master mix was added to 96‐well filter plates. Every plate also included three peptide‐only wells for background quantification (NEG), and three wells with a positive control sample (POS) for the downstream calibration across all plates. The POS sample was composed of a pool of multiple peanut allergic subjects that were not part of this study and serves as a standard control for all BBEA runs; the POS sample is independent of the clinical or any other sample run on the plate. After 3 washes, 100 μl/well of 1:10 diluted plasma or serum samples were added in triplicates and incubated with the peptides for 2 h. After two additional washes, samples were incubated with 50 μl/well of mouse anti‐human phycoerythrin (PE) conjugated IgE (Thermo‐Pierce Antibodies, Clone BE5, 1:50 dilution) for 30 min. Plates were read on a Luminex‐200 instrument (Luminex Corporation) and IgE for each sample and epitope quantified as a Median Fluorescence Intensity (MFI).

The MFI was then converted to a “calibrated” value (calMFI) through the following steps: (1) log2‐transformation, (2) assignment of zero for values below the limit of detection (LOD), and (3) adjusting for inter‐plate variability using the “correction factor.” The median MFI was transformed for each sample (*s*) and epitope‐specific IgE (*e*) as follows:

(1) $${\text{tMFI}}_{s,e}=\log_{2}\left(\text{median}\;\left(\mathrm{MF}{I_{\text{replicat}e_{1}}}_{s,e},\mathrm{MF}{I_{\text{replicat}e_{2}}}_{s,e},\mathrm{MF}{I_{\text{replicat}e_{3}}}_{s,e}\right)+1\right);$$

(2) $$\;{\text{tMFI}}_{s,e}=\left\{\begin{array}{c} {\text{tMFI}}_{s,e},\text{if}\;{\text{tMFI}}_{s,e}\geq \mathrm{LOD}\;\\ 0,\text{if}\;{\text{tMFI}}_{s,e}<\mathrm{LOD}\; \end{array}\right.,$$

where LOD is a limit of detection of 2.4 for all epitopes. The LOD value was determined in a separate set of experiments using serial dilutions of samples with low peanut sIgE. Next, to ensure values were comparable across plates, an epitope‐specific “correction factor” was determined using the POS sample on each plate. This “correction factor” was calculated for each plate as the positive control sample (*tMFI*_*_POS*_ *)* on each plate divided by the median of positive controls (tMFI__POS_) on plates used in the Discovery phase, specifically BOPI and OPIA studies (Table S1). A calibrated MFI was then computed as follows:

(3) $$\;{\text{calMFI}}_{s,e}={\text{correction factor}}_{e}*{\text{tMFI}}_{s,e}$$

### 2.3 Determination and evaluation of the prediction rule

For each pair of ses‐IgEs (2016 combinations), a linear regression was fitted to predict the natural log of cumulative reactive dose (CRD) corresponding to CTDs of the Discovery cohort. Pearson (*r*) and Spearman (rho) correlations were used to measure the linear correlation between the score predicted by each ses‐IgE pair and the actual CRD levels of the patients. The best model was identified, which included IgE to Ara h 2_008 and Ara h 3_100 epitopes. This model was documented and locked; then its performance was evaluated on the Validation samples. Additionally, based on the model's predictor values, patients were split into 3 groups of dose‐reactivity: “low,” “moderate,” or “high” using the following boundaries where a stepwise increase was observed [−∞, 5.34), [5.34, 6.38], (6.38, ∞]. Within each group, the proportion of subjects that reacted at 4, 14, 44, 144, 444, and 1444 mg of peanut protein was calculated; 1000 bootstrap simulations were used to estimate 95% confidence intervals (CI). Study schematic is outlined in Figure S1.

Amino acid sequences of Ara h 2_008 and Ara h 3_100 epitopes were mapped to the conformational structures of *Ara h 2* (3OB4) and *Ara h 3* (3C3V) proteins. However, 6/15 amino acids for Ara h 2_008 and all 15 for Ara h 3_100 were missing from those structures. They were then reconstructed using Swiss‐Model (https://swissmodel.expasy.org/; Q6PSU2 for Ara h 2 and B5TYU1 for Ara h 3) and visualized using the PyMOL software.

### 2.4 Statistical analyses

CTD values were normalized using natural logarithm (ln). CalMFI values for all 64 ses‐IgEs for patients in the Discovery cohort are presented in a heatmap as a z‐score for each epitope. Using these z‐scores, for each patient we computed a number of IgE‐binding epitopes, that is an epitope is considered “recognized” if z‐score >0. Spearman correlations among ses‐IgEs are presented, with *p*‐values <.00078125 considered significant (Bonferroni correction for 64 tests).

## 3 RESULTS

### 3.1 Study design and cohorts

**FIGURE 1:**
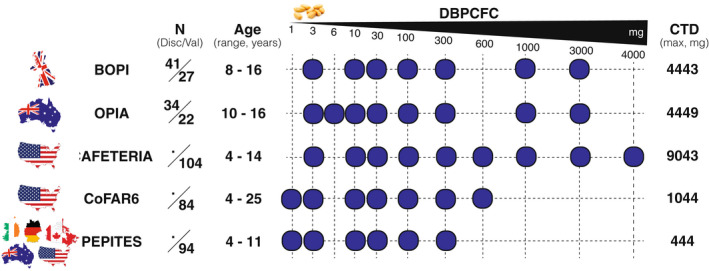
Study samples and DBPCFC dosing (Disc, Discovery; Val, Validation).

Four hundred and six peanut‐allergic subjects from 5 independent cohorts covering multiple countries, who underwent DBPCFC were included in this study: BOPI (*n* = 68), OPIA (*n* = 56), CAFETERIA (*n* = 104), CoFAR6 (*n* = 84), and PEPITES (*n* = 94, Figure 1). Participants' ages ranged across cohorts from 4 to 25 years of age. The DBPCFC protocols varied by study but followed PRACTALL guidelines for semi‐log incremental dose increase.^23^

In adherence with the guidelines by the National Academy of Medicine, the development of the CTD algorithm was done in two phases: Discovery and Validation. Seventy‐five subjects from BOPI (*n* = 41) and OPIA (*n* = 34) were randomly assigned to the Discovery cohort. The validation cohort included 201 subjects from CAFETERIA, CoFAR6, and the remaining BOPI (*n* = 27), and OPIA (*n* = 22) participants. Additionally, all the PEPITES' subjects were used for a final testing of the algorithm.

### 3.2 Ses‐IgEs are associated with CTDs

**FIGURE 2:**
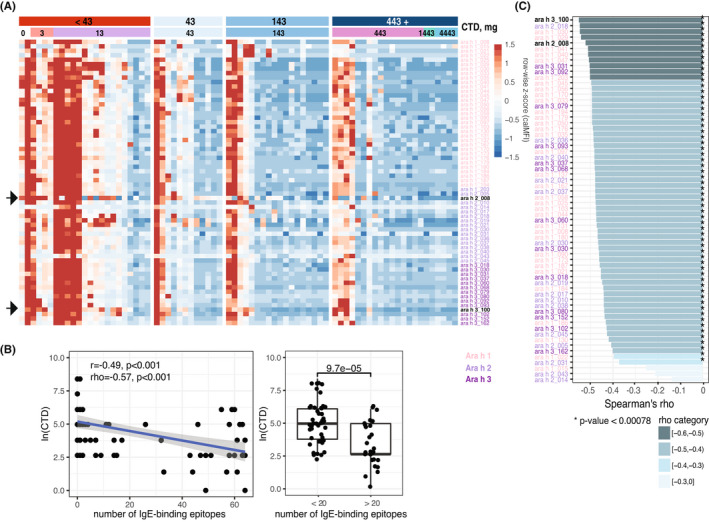
Ses‐IgE association with the CTD. (A) Patient‐specific heatmap with samples as columns and 64 ses‐IgEs as rows; represented as row‐wise z‐score of CalMFI (red – high level, blue – low). (B) Association of CTD (natural log) and a number of IgE‐binding epitopes for each subject as a scatterplot (left) or boxplot (right, Wilcoxon rank sum test *p*‐value). (C) Correlation of each of the 64 ses‐IgE with the CTD (color represents rho coefficient's magnitude, star if the *p*‐value is significant after Bonferroni correction).

Sixty‐four ses‐IgEs were evaluated in 75 subjects in the Discovery cohort (Figure 2A), with patients reacting at lower CTDs generally having a greater number of epitopes recognized by IgE antibodies (Figure 2B). Several studies have demonstrated that IgE diversity (recognition of a greater number of epitopes, i.e., “epitope spreading”) is associated with adverse outcomes, that is, more severe allergic reactions or a persistent disease phenotype.^14^, ^15^, ^24^ We observed a moderate negative correlation between the number of IgE epitopes and CTDs (rho = −0.57, *p* < .001). This means that higher IgE diversity is associated with a lower amount of peanut that a patient can consume without experiencing allergic symptoms. When we binarized a number of IgE‐binding epitopes (using the natural split observed on a scatterplot as shown in Figures 2B and S2), there was a significant association between IgE recognition of more than 20 (31%) epitopes and having lower CTDs (*p* < .001).

Individual ses‐IgE antibodies had strong pairwise correlation between themselves (median rho = 0.85 [0.76, 0.87]), as we have observed previously.^25^ Of all ses‐IgEs, 61 (95%) were negatively correlated with CTDs (Figure 2C). However, those association were variable, ranging from rho = −0.14 to −0.55, indicating that IgE‐binding epitopes have varying impact on the amount of peanut a patient can consume. It is plausible to assume that a combination of at least two such epitopes could be a stronger predictor of CTD.

### 3.3 Ses‐IgE‐based algorithm predicts peanut threshold doses

We identified a pair of ses‐IgEs that together provided the best prediction of CRD (Table S2), and devised a prediction rule for each sample *(s)* using only the Discovery cohort:

$$\begin{array}{c} \text{Predicted Score}\left(s\right)=6.83-0.23*{\text{calMFI}}_{\text{Ara}\;h\;2\_008\text{(s)}}\\ \;-0.13*{\text{calMFI}}_{\text{Ara}\;h\;3\_100\text{(s)}} \end{array}$$

**FIGURE 3:**
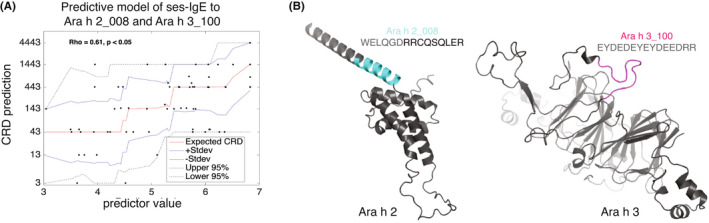
Discovery of the ses‐IgE predictor. (A) Association of the ses‐IgE predictor and CRD in the Discovery cohort showing step‐like increases (red line – expected value, blue lines ±1 standard deviation, dotted lines – 95% confidence interval). (B) Conformational structure of the two IgE epitopes on their respective proteins.

The correlation of the predicted score with the actual dose was rho = 0.61 (*p* < .001) in the Discovery cohort, which is higher than correlations of individual antibodies (Figure 2C). Importantly, the predicted score increased incrementally with the increase in CRD (Figure 3A).

This algorithm was then documented, locked, and validated on samples and outcome data from 201 subjects which had not been used in any aspect of the discovery process. The performance in this set of previously “unseen” subjects, as expected, was lower, with Spearman correlation of 0.51 (*p* < .001).

The two IgE‐binding epitopes that were included in the algorithm were from *Ara h 2* and *Ara h 3* proteins: Ara h 2_008 and Ara h 3_100. The amino acid sequences and positions on reconstructed conformational proteins for both epitopes are shown on Figure 3B.

### 3.4 Patient stratification based on ses‐IgE predictor provides reaction probabilities

**FIGURE 4:**
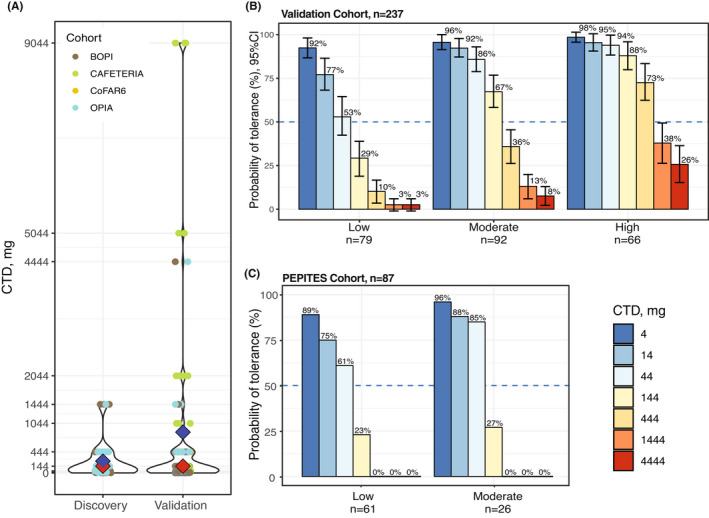
Dose‐reactivity groups and probabilities of tolerance. (A) Distribution of CTDs shown as a violin plot, colored by four cohorts (blue diamond – mean, red – median). (B) Bar chart of probabilities with 95% CI of tolerance at each peanut dose for “low”, “moderate”, and “high” dose‐reactivity groups in 237 Validation subjects. (C) Bar chart of probabilities of tolerance at each peanut dose for “low” and “moderate” reactivity groups in 87 PEPITES subjects (only seven subjects were assigned to the “high” group, not shown). In PEPITES, the maximum dose administered during DBPCFC was 300 mg.

Since CTD values are not truly continuous, generally with threefold increases at each of the escalation doses, and the sample size at each dose tends to be small (Figures 4A, Table S3), a large number of subjects will need to undergo DBPCFCs to devise a predictive rule with high correlation with CTDs. To address this limitation and to allow flexibility for our predictive algorithm as more data become available, subjects were separated into three groups of dose reactivity: “low” (*n* = 79), “moderate” (*n* = 92), and “high” (*n* = 66).

For each group, we calculated the proportion of subjects that would tolerate different doses of peanut protein (Figures 4B and S3 **)**. On average, subjects in the “high” dose‐reactivity group were 4 times more likely to tolerate a specific dose, compared with the “low” dose group. For example, predicted probabilities of tolerating 4, 14, 44, 144, and 444 mg in the “low” group were 92%, 77%, 53%, 29%, 10% compared with 98%, 95%, 94%, 88%, 73% in the “high” dose‐reactivity group.

As an additional validation step of these results, we applied the ses‐IgE‐based algorithm to the PEPITES subjects to obtain their probability distributions. The PEPITES cohort differed from the rest in that all 94 patients had CTDs less than 144 mg, as per eligibility criteria. Ninety‐three percent of the subjects were assigned to either “low” or “moderate” ses‐IgE groups, suggesting that they would have high probability of reacting at low doses, and the distributions of the CTDs in those two groups were similar to that derived from the 237 validation subjects (Figure 4C).

## 4 DISCUSSION

DBPCFCs are the “gold standard” for diagnosing food allergy, helping establish reactivity thresholds, and determining if this threshold changes over time or as a result of an intervention.^12^ This information can be very valuable to allergic patients and their caregivers, as it suggests the stringency of avoidance that they might observe, based upon an individualized risk assessment. For example, if a patient reacts to a 1000 mg dose of peanut protein, then it is unlikely they would have an objective reaction to 100 mg peanut protein (equivalent to around half a peanut kernel); this information can thus inform the degree of peanut avoidance required and reduce anxiety. DBPCFCs are also key in assessing the impact of potential disease‐modifying interventions such as immunotherapy; as the latter becomes more mainstream in clinical practice. However, DBPCFCs are less pragmatic and even open‐label OFCs with fewer doses might be considered impractical. Food challenges in food‐allergic individuals are not without risk, and therefore, require trained personnel and proper equipment, as well as causing inconvenience to patients and their families. Our goal was to develop a test that can predict peanut threshold amounts, thus providing more information to both patients and providers and informing, if not reducing the need for food challenges.

Development of any diagnostic test requires a priori randomization of subjects into discovery and validation sets.^11^ Regression models, similar to other machine learning algorithms, tailor the predictions to the data they are derived from, where the “learning” could include random noise. This way, it is possible to have almost perfect predictions if the model is complex enough (e.g., many predictors and polynomial terms). However, when such a model is employed on a new set of observations, the performance will dramatically decrease, indicating the “overfitting” of the model. Therefore, it is important to always have a separate set of subjects, when sample size allows, or use appropriate resampling techniques to obtain generalizable performance metrics. Since a new set of subjects will have different variability, it is common to see some drop in performance in the validation cohort, which gives an estimate of how the algorithm will perform in “real world”. Ideally, a validation set should consist of a population that was not part of the discovery set to ensure accurate representation of external validity. In this work, we have obtained samples from five independent cohorts from five countries: Australia, UK, US, Ireland, and Germany. All the development work, including descriptive statistics, was carried out in the Discovery subjects, and only after the final algorithm was locked and documented was it analyzed on the Validation cohort.

We observed that both the levels of individual ses‐IgEs, as well as IgE epitope diversity were inversely associated with the CTD. Allergic effector cells, that is, basophils and mast cells, are saturated with high‐affinity IgE (FceRI) receptors, which upon allergen exposure, IgE molecules on the surface of those cells cross‐link, leading to the release of immune mediators.^26^ Higher levels of IgE in serum/plasma correlate with the number of antibodies on the cell surface,^27^ and higher IgE diversity may result in antigen (peanut protein) being more readily detectable, leading to allergic reactions. While we observed moderate correlations, we hypothesized that a combination of several ses‐IgE antibodies could have a stronger association with the CTD.

We set out to develop a predictor using a machine learning approach to evaluate all pairwise ses‐IgE combinations, until the best pair was identified. Two antibodies with the combined strongest association were specific to the Ara h 2_008 and Ara h 3_100 epitopes. Interestingly, the algorithm did not select a pair of IgE epitopes that individually had the highest correlation with the CTD, suggesting an additive effect of these two markers. These IgE epitopes were previously identified as important early predictors of peanut allergy development^22^ and were detected in more allergic compared to sensitized only subjects.^28^ Ara h 2_008 was identified as a main diagnostic IgE epitope for peanut allergy^13^ and showed greater increases over time in children who developed peanut allergy in the avoidance arm of the LEAP trial.^21^

The CTD predictions were limited by the sample size, since dose increments, in general, follow semi‐log increases and not every dose is equally represented. Additionally, while all the studies followed PRACTALL guidelines, study‐specific dose variations were still present. These factors make CTD predictions less reliable; to address this limitation and make sure that the algorithm can get more precise as more data become available, the outcome of the test was designed to provide a specific probability of reactions at all CTDs. Using the predicted score, patients were assigned to “low,” “moderate,” or “high” dose‐reactor groups, and the probability of tolerating any given dose was fourfold different between the “high” and “low” groups.

This risk group assignment is valuable for many purposes, including deciding whether a patient should undergo an OFC to confirm a safe tolerated dose or whether they should maintain stringent allergen avoidance and/or pursue oral immunotherapy (OIT), and to monitor possible allergy resolution over time. For example, a subject with “low” dose‐reactivity group could benefit from OIT.^29^ Even though OIT requires considerable time, effort, and risk of adverse reactions, the benefits for someone with a low tolerance threshold would likely outweigh the burden of lifestyle change required to undergo OIT; while someone in “high” dose‐reactivity group wishing to pursue OIT could initiate OIT at a higher dose, thus shortening the time necessary to achieve the maintenance dose. Additionally, subjects in the “moderate” or “high” groups may consider undergoing a single‐dose (one shot) OFC of peanut protein to confirm their tolerance, and thus, allow for a less stringent avoidance regimen, that is, consumption of foods with precautionary allergen labeling. For example, a patient in the “high” group may wish to undergo a one‐dose challenge to 100 or 300 mg of peanut protein to confirm tolerance at these levels since there would be a 4 out of 5 or 3 out of 4 chance of them tolerating these doses, respectively. Similarly, a patient in the “moderate” group may wish to try a one‐dose challenge to 30 or 100 mg since they would have a 4 out of 5 or 2 out of 3 chance of tolerating these doses. While incorporating low‐doses of peanut‐containing products would not be recommended, an understanding that low‐dose contamination of food would not likely lead to an allergic reaction could reduce anxiety significantly and lead to a marked improvement in quality of life.

As all samples and outcomes in this study were from the baseline visit of clinical trials, and the subjects were avoiding peanut, this algorithm would need further evaluation to determine its utility for subjects undergoing OIT, where other immune factors could affect the results (e.g., generation of allergen‐specific IgG and IgA antibodies).^30^ Thresholds established with the BBEA are based on the outcomes of oral food challenges performed under ideal, steady‐state conditions. Therefore, patients should be advised that certain co‐factors, for example, exercise, NSAIDs, alcohol, high fever, heavy pollen season, may influence the predicted reaction threshold. The strengths of this algorithm are its development and validation on separate cohorts, spanning multiple countries and a wide age range (4–25 years); the use of a small amount of serum/plasma sample (< 20 ul, can be used from frozen); and the possibility for further refinement as more data become available.

In conclusion, this is a first validated algorithm using peanut‐specific epitopes to predict probabilities of reaction to different amounts of peanut in allergic subjects and may provide a useful surrogate for peanut food challenges.

## AUTHOR CONTRIBUTIONS

The study was conceptualized by P.K., R.G., M.S., and H.S. Sample acquisition and data curation was carried out by C.H., H.B., P.T., and D.K. Data were analyzed and visualized by M.S., P.K., C.H., and H.B; P.T., D.K., S.H.S. were site PIs for BOPI, OPIA, and CAFETERIA studies and provided information and advice regarding participant characterization. M.S. and H.S wrote the original draft. All authors reviewed, edited, and approved the manuscript.

## FUNDING INFORMATION

The study was funded in part by a grant from National Institute of Allergy and Infectious Diseases (NIH‐NIAID AI‐066738, U19AI066738, U01AI066560, and U19AI136053), the David H. and Julia Koch Research Program in Food Allergy Therapeutics, and AllerGenis LLC. Food challenges in the BOPI study were funded through a UK Medical Research Council Clinician Scientist award to PJT (reference MR/K010468/1).

## CONFLICT OF INTEREST

Maria Suprun has nothing to disclose. Paul Kearney, Clive Hayward, Heather Butler, and Robert Getts are employees of AllerGenis LLC. Dr. Getts has a patent PCT/US15/020715 (WO) pending. Scott H Sicherer reports royalty payments from UpToDate and from Johns Hopkins University Press; grants to his institution from the National Institute of Allergy and Infectious Diseases, and from Food Allergy Research and Education; and personal fees from the American Academy of Allergy, Asthma and Immunology as Deputy Editor of the Journal of Allergy and Clinical Immunology: In Practice, outside of the submitted work. Paul J. Turner reports grants from UK Medical Research Council, NIHR/Imperial Biomedical Research Centre and Jon Moulton Charity Trust; personal fees from UK Food Standards Agency, Aimmune Therapeutics, Allergenis, Aquestive Therapeutics, outside the submitted work. Dianne E Campbell is a part‐time employee of DBV Technologies and reports receiving grant support from National Health and Medical Research Council of Australia and personal fees from Allergenis (Advisory board) and Westmead Fertility Centre (Governance committee). Hugh A Sampson reports grants from Immune Tolerance Network, and NIH‐NIAID, consulting fees from DBV Technologies, N‐Fold, LLC, and Siolta Therapeutics; Mount Sinai has licensed the technology for a bead‐based epitope assay for food‐allergen epitope analysis to AllerGenis LLC, where Dr. Sampson serves as an unpaid Board of Directors member and advisor.

## Supporting information


Appendix S1
