# Supplementary material for: Predicting probability of tolerating discrete amounts of peanut protein in allergic children using epitope‐specific IgE antibody profiling
Source: Allergy. 2022 Aug 17;77(10):3061–9. doi: 10.1111/all.15477 (PMC10286745; doi:10.1111/all.15477)

**Title:** Predicting probability of tolerating discrete amounts of peanut protein in allergic children using epitope-specific IgE antibody profiling

**Authors:** Maria Suprun, PhD ^1^, Paul Kearney, PhD ^2^, Clive Hayward, BASc ^2^, Heather Butler, MS ^2^, Paul J. Turner, PhD ^3^, Dianne E Campbell, PhD^4^, Robert Getts, PhD ^2^, Hugh A. Sampson, MD ^1^

Table of Contents:

[Supplementary Table 1 2](#_Toc103696198)

[Supplementary Table 2 3](#_Toc103696199)

[Supplementary Table 3 4](#_Toc103696200)

[Supplementary Figure E1 5](#_Toc103696201)

[Supplementary Figure E2 6](#_Toc103696202)

[Supplementary Figure E3 7](#_Toc103696203)

# Supplementary Table 1

Summary of “correction factor” values for ses-IgEs to Ara h 2_008, Ara h 3_100, and average of all 64 epitopes, separate for serum and plasma plates.

|  | **Plasma** | |
| --- | --- | --- |
| **Epitope** | **Average** | **Standard deviation** |
| Ara h 2_008 | 1.025 | 0.047 |
| Ara h 3_100 | 1.023 | 0.050 |
| All 64 ses-IgEs | 1.017 | 0.052 |
|  |  |  |

|  | **Serum** | |
| --- | --- | --- |
| **Epitope** | **Average** | **Standard deviation** |
| Ara h 2_008 | 1.003 | 0.0142 |
| Ara h 3_100 | 1.008 | 0.0147 |
| All 64 ses-IgEs | 1.003 | 0.056 |

# Supplementary Table 2

Performance of the top 10 models as a Spearman’s correlation of the predictor score and actual outcome.

| **Epitope_1** | **Epitope_2** | **Correlation** |
| --- | --- | --- |
| Ara h 2_008 | Ara h 3_100 | 0.59782799 |
| Ara h 1_173 | Ara h 3_100 | 0.59246847 |
| Ara h 1_030 | Ara h 3_100 | 0.58229 |
| Ara h 3_080 | Ara h 3_100 | 0.58216939 |
| Ara h 2_019 | Ara h 3_100 | 0.58180186 |
| Ara h 2_021 | Ara h 3_100 | 0.58079298 |
| Ara h 1_045 | Ara h 3_100 | 0.58074332 |
| Ara h 2_037 | Ara h 3_100 | 0.57868439 |
| Ara h 3_102 | Ara h 3_100 | 0.57834018 |
| Ara h 1_187 | Ara h 3_100 | 0.57758219 |

# Supplementary Table 3

Number of samples at each CRD in the Discovery cohort.

| **BOPI** |  |
| --- | --- |
| **CRD** | **# Samples** |
| 3 | 2 |
| 13 | 4 |
| 43 | 12 |
| 143 | 7 |
| 443 | 6 |
| 776 | 1 |
| 1443 | 4 |
| 4443 | 1 |
| >4443 | 4 |
|  |  |
| **OPIA** |  |
| **CRD** | **# Samples** |
| 3 | 0 |
| 9 | 0 |
| 19 | 0 |
| 49 | 5 |
| 149 | 4 |
| 179 | 1 |
| 449 | 12 |
| 549 | 2 |
| 749 | 2 |
| 1449 | 7 |
| 4449 | 1 |

# Supplementary Figure E1

Schematic outline of the study.


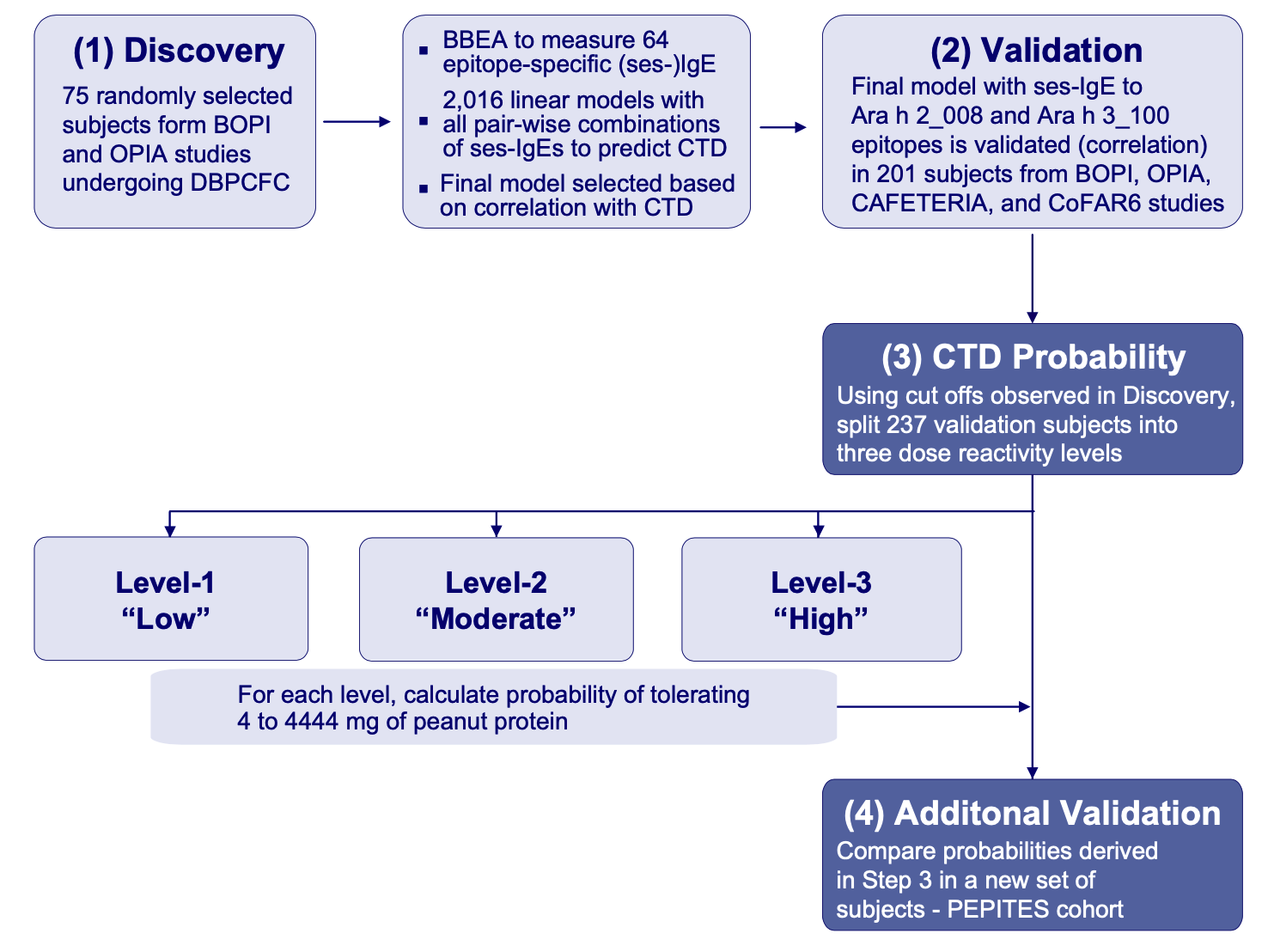


# Supplementary Figure E2

Association of CTD (natural log) and a number of IgE-binding epitopes for each subject as a boxplot using 20, 30, or 40 as cut off values (Wilcoxon rank sum test p-value).


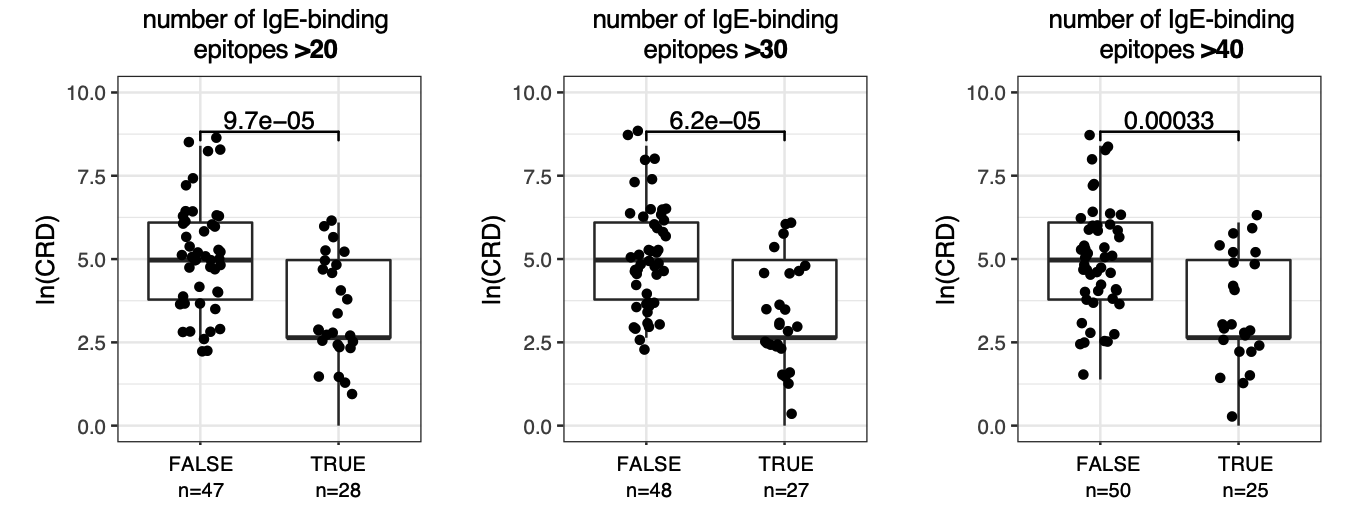


# Supplementary Figure E3

Bar charts with probabilities of tolerance at each CTD for “low”, “moderate”, and “high” dose-reactivity groups by Validation cohorts.


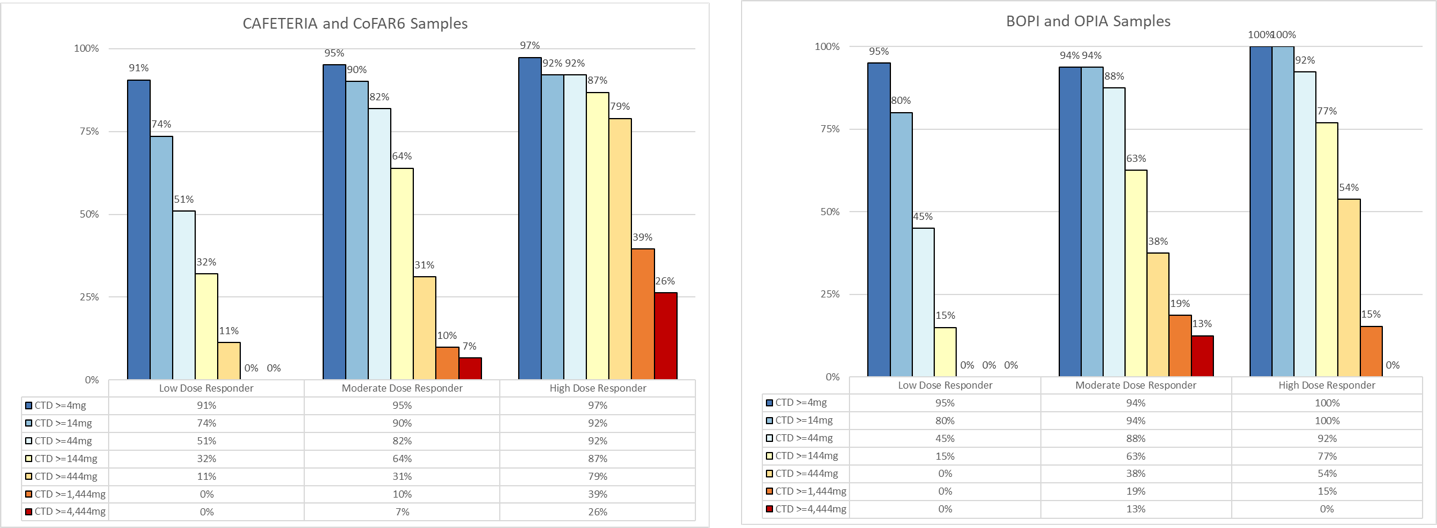

Supplement: Supplementary file 1 — Appendix S1 [file ALL-77-3061-s001.docx]
